# A genome-wide association study reveals novel loci and candidate genes associated with plant height variation in *Medicago sativa*

**DOI:** 10.1186/s12870-024-05151-z

**Published:** 2024-06-13

**Authors:** Xueqian Jiang, Tianhui Yang, Fei He, Fan Zhang, Xu Jiang, Chuan Wang, Ting Gao, Ruicai Long, Mingna Li, Qingchuan Yang, Yue Wang, Tiejun Zhang, Junmei Kang

**Affiliations:** 1 Institute of Animal Science, Chinese Academy of Agricultural Sciences, Beijing, China; 2 School of Grassland Science, Beijing Forestry University, Beijing, China; 3 Institute of Animal Science, Ningxia Academy of Agricultural and Forestry Sciences, Yinchuan, Ningxia China; 4 Institute of Grassland Research, Chinese Academy of Agricultural Sciences, Hohhot, Inner Mongolia China; 5 Beijing NO.19 High School, Beijing, China

**Keywords:** Alfalfa (*Medicago sativa*), GWAS, Plant height, Phosphate transporter

## Abstract

**Background:**

Plant height (PH) is an important agronomic trait influenced by a complex genetic network. However, the genetic basis for the variation in PH in *Medicago sativa* remains largely unknown. In this study, a comprehensive genome-wide association analysis was performed to identify genomic regions associated with PH using a diverse panel of 220 accessions of M. sativa worldwide.

**Results:**

Our study identified eight novel single nucleotide polymorphisms (SNPs) significantly associated with PH evaluated in five environments, explaining 8.59–12.27% of the phenotypic variance. Among these SNPs, the favorable genotype of *chr6__31716285* had a low frequency of 16.4%. *Msa0882400*, located proximal to this SNP, was annotated as phosphate transporter 3;1, and its role in regulating alfalfa PH was supported by transcriptome and candidate gene association analysis. In addition, 21 candidate genes were annotated within the associated regions that are involved in various biological processes related to plant growth and development.

**Conclusions:**

Our findings provide new molecular markers for marker-assisted selection in *M. sativa* breeding programs. Furthermore, this study enhances our understanding of the underlying genetic and molecular mechanisms governing PH variations in *M. sativa*.

**Supplementary Information:**

The online version contains supplementary material available at 10.1186/s12870-024-05151-z.

## Introduction

Plant height (PH) is a key trait of plant architecture that influences biomass production [1, 2], grain yield [3], and lodging resistance [4]. PH is governed by various factors, primarily genes involved in the biosynthesis or signaling of gibberellin (GA) and brassinolide (BR), two plant hormones [5–7]. For example, the *semi-dwarf1* gene (*SD1*) in rice, which encodes GA20-2 oxidase, a key enzyme in the GA biosynthetic pathway, is widely used to breed modern semi-dwarf rice varieties [8, 9]. In chrysanthemum (*Chrysanthemum morifolium*), PH is directly regulated by the YAB transcription factor *CmDRP*, which represses the expression of *CmGA3ox1*, a gene encoding GA3 oxidase, by binding to its promoter [10]. Rice height is also regulated by BR signaling and metabolism in the lower internodes, where OSH15 forms a protein complex (DLT-OSH15) with DLT and binds to the promoter of *OsBRI1*, a gene encoding the BR receptor [11]. Other plant hormones, such as auxin, cytokinin, and ethylene, also modulate PH in addition to GA and BR [5, 6]. For example, *very long-chain fatty acids* (*VLCFAs*) can induce the expression of genes related to ethylene synthesis, which in turn affects rice height [12]. In maize, PH can be reduced by overexpressing *ZmPIN1a*, which enhances the transport of IAA, the auxin hormone, from the shoot to the root [13].

In contrast to annual crops such as rice and maize, alfalfa (*Medicago sativa*), a perennial forage legume, has a deep crown and a long taproot system [14]. The genetic regulation of PH in alfalfa is poorly understood, but may involve different hormonal pathways. Alfalfa is the most widely cultivated perennial legume forage worldwide [15], and has a long history of use in China. It is known as the “Queen of Forages” because of its high yield, rich nutrition, high protein content, and good palatability [15]. China is the second largest alfalfa producer after the United States [16]. Nevertheless, the growing demand for the dairy and animal husbandry industries in China is hindered by the insufficient domestic supply of alfalfa forage [17]. Consequently, China is highly dependent on importing substantial amounts of alfalfa hay, primarily from the USA [18]. In order to address this issue, Chinese alfalfa breeders are actively working towards developing domestic cultivars that demonstrate exceptional productivity and adaptability across various ecological regions. PH serves as a key phenotypic trait for artificial selection in breeding programs, as it exhibits a significant positive correlation with forage yield [19]. Increasing PH can effectively improve biomass yield. The identification and characterization of genes associated with PH can greatly facilitate the development of new alfalfa varieties with improved yield. This will be of immense benefit to alfalfa breeders, who are increasingly challenged by protein shortages due to the expanding global population.

Genome-wide association studies (GWAS) have emerged as a cost-effective and efficient tool for dissecting quantitative trait loci (QTL) and candidate genes related to complex agronomic traits within *M. sativa*, and have been widely used in recent years [20–23]. Compared to conventional linkage mapping methods, GWAS boasts enhanced resolution and ability to identify smaller effect QTLs. Yu (2017) identified 28 QTLs associated with biomass yield under well-watered conditions and 4 QTLs under water deficit conditions in a panel of 200 accessions of *M. sativa* [21]. Liu and Yu (2017) detected 33 and 14 loci significantly associated with dry weight and PH under salt stress conditions, respectively [22]. Moreover, GWAS serves as a robust avenue to identify functional and agronomic genes. Sakiroglu and Brummer (2017) identified five candidate genes that influence plant growth in diploid accessions of *M. sativa* using genotyping-by-sequencing based GWAS [23]. Wang et al. (2019) found a significant SNP (*rs12428*) located within the *MTR_2 g105090* gene on chromosome 2 in *M. sativa* associated with variation in PH and explaining 12.6% of the phenotypic variation. This SNP was further validated to modulate PH by overexpressing *MTR_2 g105090* in *Arabidopsis thaliana* [20]. Consequently, GWAS has been successfully applied to dissect QTLs and candidate genes for complex quantitative traits in alfalfa, thus facilitating the understanding of the molecular mechanisms underlying PH and other important traits in alfalfa and accelerating the breeding process for high-yielding varieties.

PH is a key agronomic trait influenced by a complex genetic network. Although some QTLs and SNPs associated with PH have been reported [19, 20, 22, 24, 25], the genetic basis for the variation in PH in alfalfa remains largely unknown. In this study, the PHs of 220 diverse alfalfa accessions were measured of different origins and improved statuses in five distinct environments. A total of 875,023 high-quality SNPs from previous studies were used to perform a comprehensive genome-wide association study to detect novel SNPs and candidate genes associated with PH variation in alfalfa. Our results provide new insights into the genetic and molecular mechanisms underlying PH variation in alfalfa and offer valuable molecular markers for marker-assisted selection in alfalfa breeding.

## Materials and methods

### Plant materials

GWAS was performed on an association panel of 220 *M. sativa* accessions, previously reported by Long et al. (2022) [26]. The germplasm collection consisted of 26 accessions from the Medium Term Library of the National Grass Seed Resources of China and 194 accessions acquired through the U.S. National Plant Germplasm System online database (https://npgsweb.ars-grin.gov/gringlobal/ search) (Table S1). This panel represents the global genetic diversity of *M. sativa*, collected from 50 countries across six continents. The panel comprised 220 genotypes, including 16 wild accessions, 95 landraces, 55 cultivars, 30 breeding materials, and 24 accessions with unclear improvement status.

### Experimental design and phenotypic assessment

PH phenotypes from 220 accessions were collected in two locations, namely Langfang, Hebei Province (the research station of the Chinese Academy of Agricultural Sciences (CAAS), 39.59°N, 116.59°E) and Yinchuan, Ningxia Province (the research station of the Ningxia Academy of Agricultural and Forestry Sciences, 38.21°N, 106.22°E). In Langfang (LF), seeds were sown in a greenhouse in December 2017, and 15 uniform seedlings were selected and transplanted into the field in April 2018. The field experiment had a randomized block design with three replicates. Five individual plants of the same accession were spaced 30 cm apart in the field, and rows and columns among the accessions were spaced 65 cm apart. In Yinchuan (YC), 220 genotype seeds germinated in a greenhouse in December 2018 and were transplanted to the field in May 2019. The field experiment had a randomized block design with three replicates and three individual plants in each replicate. The distance between rows and columns was 70 cm, and the plant-to-plant distance was 40 cm.

The phenotypes were measured in 2019, 2020, and 2021 in LF and in 2020 and 2021 in YC. PH was defined as the length of the longest branch from the soil to the tip of the branch at the beginning of the flowering stage. Phenotypic data was collected in five environments (LF19, LF20, LF21, YC20, and YC21). The R package *psych* was used to perform a statistical analysis of PH. Furthermore, the best linear unbiased estimator (BLUE) and the broad sense heritability (H^2^) of PH were estimated using the ANOVA function in the IciMapping software [27]. Our GWAS incorporated phenotypic data from these six environments, comprising both the mean values of the five environments and the BLUE value.

### Genotyping and genome-wide association study

The genotype of this panel was published in our previous study [28]. A total of 875,023 SNPs were identified in the panel after filtering for missing rate (< 0.1) and minor genotype frequency (> 0.05). The SNP-based population structure analysis divided the 220 accessions into three subgroups [29]. Subgroup A consisted of accessions from the presumed centers of the geographical origin of the species. Subgroup B consisted mainly of accessions from China, while subgroup C included accessions from America and Europe. GWAS of PH was performed using Tassel 5.0 with the general linear model (GLM) [30]. And the genome-wide significance threshold, -log_10_(*P*), was set at 6.0 (GLM model). For reducing the likelihood of false positive results, the mixed linear model (MLM), compressed mixed linear model (CMLM) in Tassel 5 [30], and the Farm-CPU model in GAPIT 3.0 were also used for performing the GWAS [31, 32]. If a significant SNP identified by the GLM model was also identified by any other method with a threshold greater than 4, it was considered a strong association signal and used for further analysis. Principal component analysis (PCA) and kinship analysis (K) were computed in Tassel and utilized for association analysis. The R package *CMplot* was used to draw a Manhattan plot to display the results of the association analysis.

### Analysis of favorable haplotypes by GWAS

Significant SNPs linked to PH have been identified via GWAS. T-tests were conducted to ascertain favorable haplotypes by evaluating the connection between various haplotypes and PH. Favorable haplotypes in this research were characterized by greater PH. In addition, by analyzing the correlation between PH and the number of favorable haplotypes carried by each accession in the association panel, explore the potential use of these favorable haplotypes in improving alfalfa PH.

### RNA-seq data analysis

The RNA-seq data from different alfalfa tissues (leaves, roots, nodules, flowers, elongation internodes, and post-elongation internodes) were obtained in a previous study [33]. Genes differentially expressed in the elongation internodes (ES) and post-elongation internodes (PES) were considered as candidate genes that could affect the development of the alfalfa internodes. The RNA-seq data was analyzed using the pipeline described in our previous study [34]. |log2 (FoldChange)| ≥ 2 and *P* ≤ 0.01 were used as thresholds to identify differentially expressed genes (DEG).

### Gene-based association study

*Msa0882400* was identified as a candidate gene associated with alfalfa PH. To validate this result, 118 individuals with genotypes different from those in our association panel were re-sequenced and generated approximately 30 GB of raw data per accession (unpublished data). A total of 33 SNPs were detected within the *Msa0882400* gene region and its 10-kb upstream range. Information on SNPs is presented in Table S4. Six cutting seedlings from each genotype were selected to measure PH in the greenhouse. The height of each plant at the early flowering stage was measured using a ruler, and the mean value for each accession was calculated for the gene-based association study.

## Result

### Analysis of phenotypic variation

**Fig. 1:**
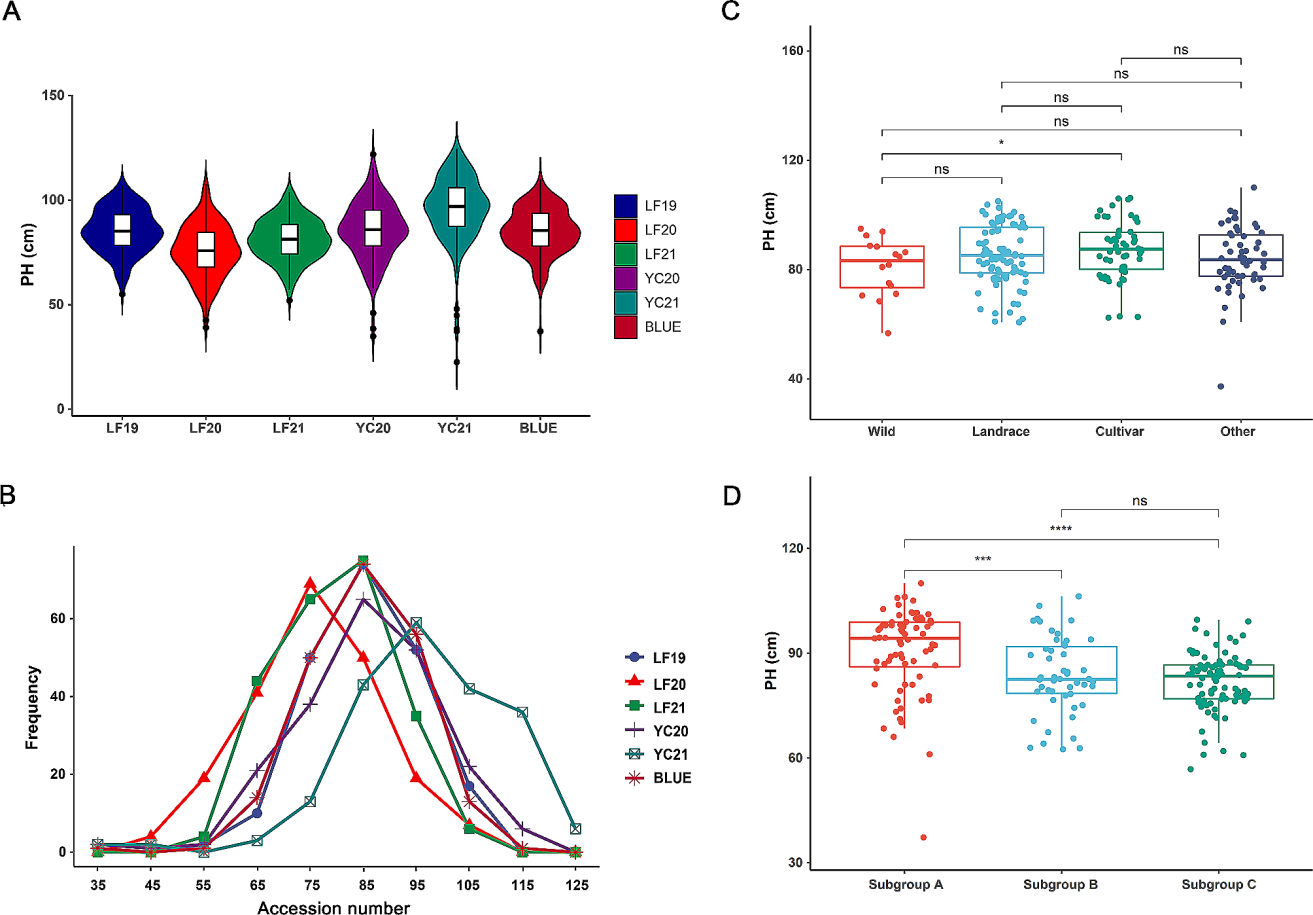
Variation in plant height across different environments and subgroups in the association panel. **A** and **B** display plant height in six environments as box plots and histograms, respectively. **C** and **D** compare plant height (BLUE values) among four subgroups based on the improvement status (wild, landrace, cultivar, and other) and three subgroups based on geographical origin (subgroup A, subgroup B, and subgroup C), respectively. The subgroup “Other” includes 30 breeding materials and 24 associations with unclear improvement status. The asterisks above the box plots in **C** and D denote significant differences between the subgroups (t-test, *P < 0.05, **P < 0.01, ****P < 0.0001, ^NS^non-significant)

The PH phenotype of the association panel varied considerably, with a phenotypic variation ranging from 12.30 to 16.76% (Table S2, Fig. 1A). The frequency distribution of PH showed a nearly normal distribution in all environments (Fig. 1B). H^2^ was 0.52, indicating that a high proportion of variability in this trait was due to environmental factors. The accessions of alfalfa grown in YC had a greater PH than those grown in LF (Table S2, Fig. 1A).

A statistical analysis of PH was performed based on breeding status and geographical origin. According to the breeding status, the cultivar accessions had significantly greater PHs than the wild accessions (t-test, *P* < 0.05). No significant differences were observed between the other subgroups (Fig. 1C). Based on our previous study, the association panel was divided into three subgroups according to geographical origin. Subgroup A comprised accessions from the presumptive geographical origin centers, while subgroups B and C comprised accessions from China and America-Europe, respectively (Table S1). There were no significant differences in PH between subgroups B and C. However, both subgroups had significantly lower PH than subgroup A (Fig. 1D). Our results suggest that there were more significant differences in PH among accessions of different geographical origins, which may indicate that geographical isolation factors play an important role in the formation of alfalfa cultivars.

### Genome-wide association study for PH

**Fig. 2:**
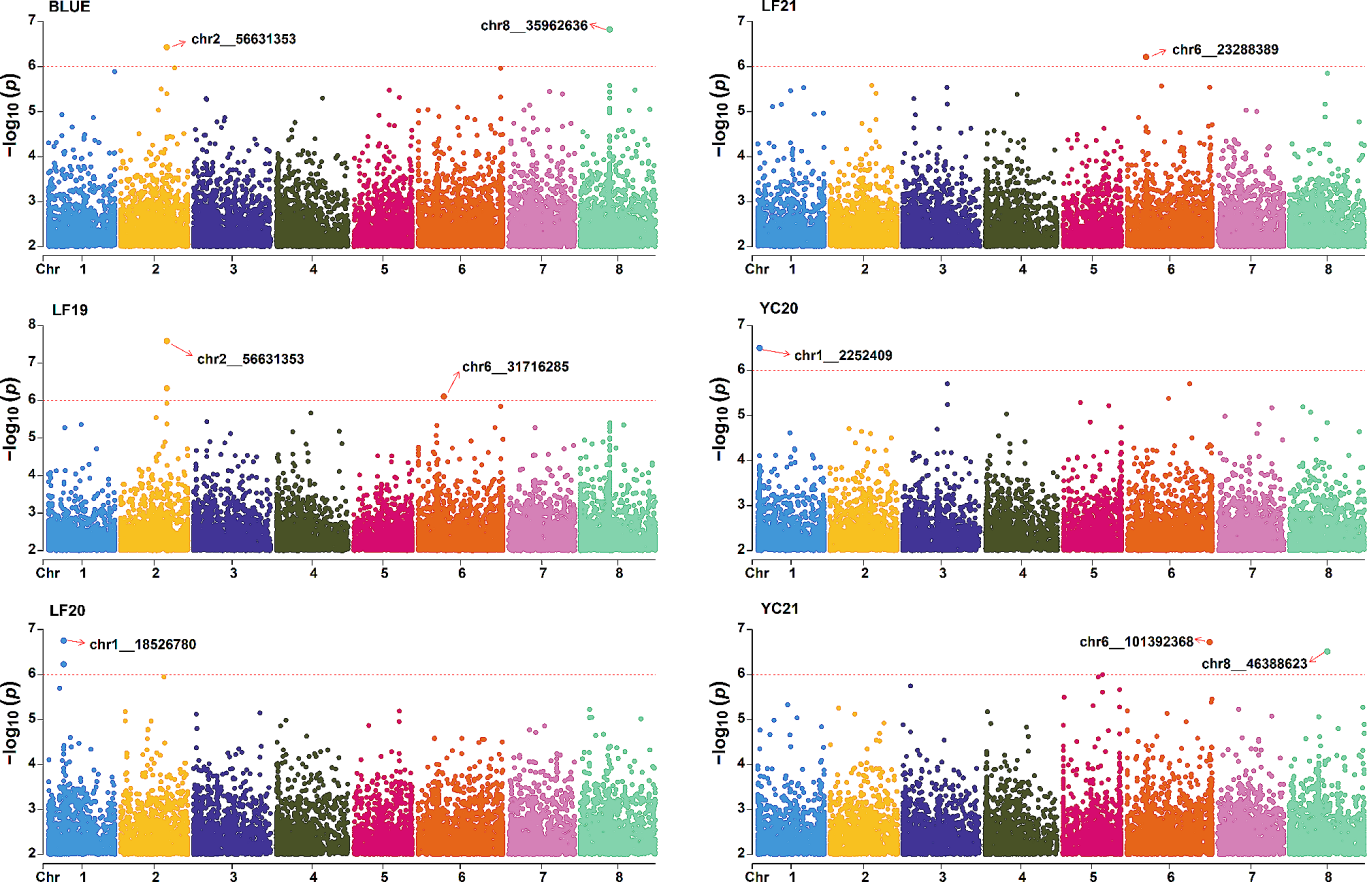
Manhattan plots of the GWAS for PH. Analysis of the genome-wide association of plant height in six environments. The SNPs marked by the red arrows were the primary SNPs, being the most significant SNPs within each QTL interval (within 1 CM)

A total of 875,023 high-quality SNPs were used to perform a GWAS for PH in *M. sativa*. Using the GLM model, ten significant SNPs (-log_10_(*P*) ≥ 6) were detected (Table 1). Among these, *chr2__56631353* was found to be significant for both BLUE and LF19. The phenotypic variance explained (PVE) by these significant SNPs ranged from 8.59 to 12.27%, and they were distributed across four chromosomes: three on chr1, two on chr2, three on chr6, and two on chr8 (Table 1; Fig. 2). For BLUE, LF19, LF20, LF21, YC20, and YC21, 2, 3, 2, 1, 1, and 2 significant SNPs were identified, respectively (Table 1; Fig. 2). The two SNPs detected by the BLUE values were located at 56.63 Mb on chr2 and 35.96 Mb on chr8 and had the highest PVEs at 12.27% and 11.59%, respectively (Table 1).

**Table 1:** Significant SNPs for plant height

Environment	SNP	Allele	Chr	Pos	-log_10_(*P*)	*R* ^2^	Favorable Allele	MLM	CMLM	FarmCPU
								-log_10_(*P*)	-log_10_(*P*)	-log_10_(*P*)
BLUE	*chr2__56631353*	G/A	2	56,631,353	6.43	12.27%	G/A	5.20	4.81	6.09
BLUE	*chr8__35962636*	A/T	8	35,962,636	6.82	11.59%	A/A	4.84	5.41	6.47
LF19	*chr2__56631353*	G/A	2	56,631,353	7.59	8.59%	G/A	6.15	4.34	7.23
LF19	*chr2__56631363*	G/A	2	56,631,363	6.33	9.10%	-	4.96	4.22	6.03
LF19	*chr6__31716285*	G/A	6	31,716,285	6.11	9.35%	G/A	5.35	**-**	6.13
LF20	*chr1__18526768*	T/G	1	18,526,768	6.23	9.05%	-	5.11	**-**	**-**
LF20	*chr1__18526780*	A/T	1	18,526,780	6.75	9.66%	A/A	5.48	**-**	**-**
LF21	*chr6__23288389*	A/G	6	23,288,389	6.21	9.08%	A/A	5.43	4.34	5.96
YC20	*chr1__2252409*	T/C	1	2,252,409	6.50	11.63%	T/C	5.98	5.29	6.43
YC21	*chr6__101392368*	C/G	6	1.01E+08	6.72	9.66%	C/C	4.77	4.69	6.41
YC21	*chr8__46388623*	A/G	8	46,388,623	6.51	9.43%	A/A	5.28	5.57	6.21

GWAS was also conducted using the MLM, CMLM (Tassel 5), and Farm-CPU (GAPIT 3.0) models to reduce the likelihood of false positive results. By comparing the SNPs detected in all four models, it was found that all SNPs detected in the GLM model except for *chr6__31716285*, *chr1__18526768*, and *chr1__18526780* were also detected in the other three models. Specifically, under the threshold (-log_10_(*P*) ≥ 4), all the significant SNPs identified by GLM were also identified by the MLM model (Table 1). Two significant SNPs (*chr1__18526768* and *chr1__18526780*) were not detected by the CMLM and Farm-CPU methods, while one significant SNP, *chr6__31716285*, was not detected by the CMLM model (Table 1). These results suggested that these SNPs identified by the GLM model were strong association signals, and more likely to have genuine associations with PH in *M. sativa*.

### The role of favorable haplotypes in alfalfa PH breeding

**Fig. 3:**
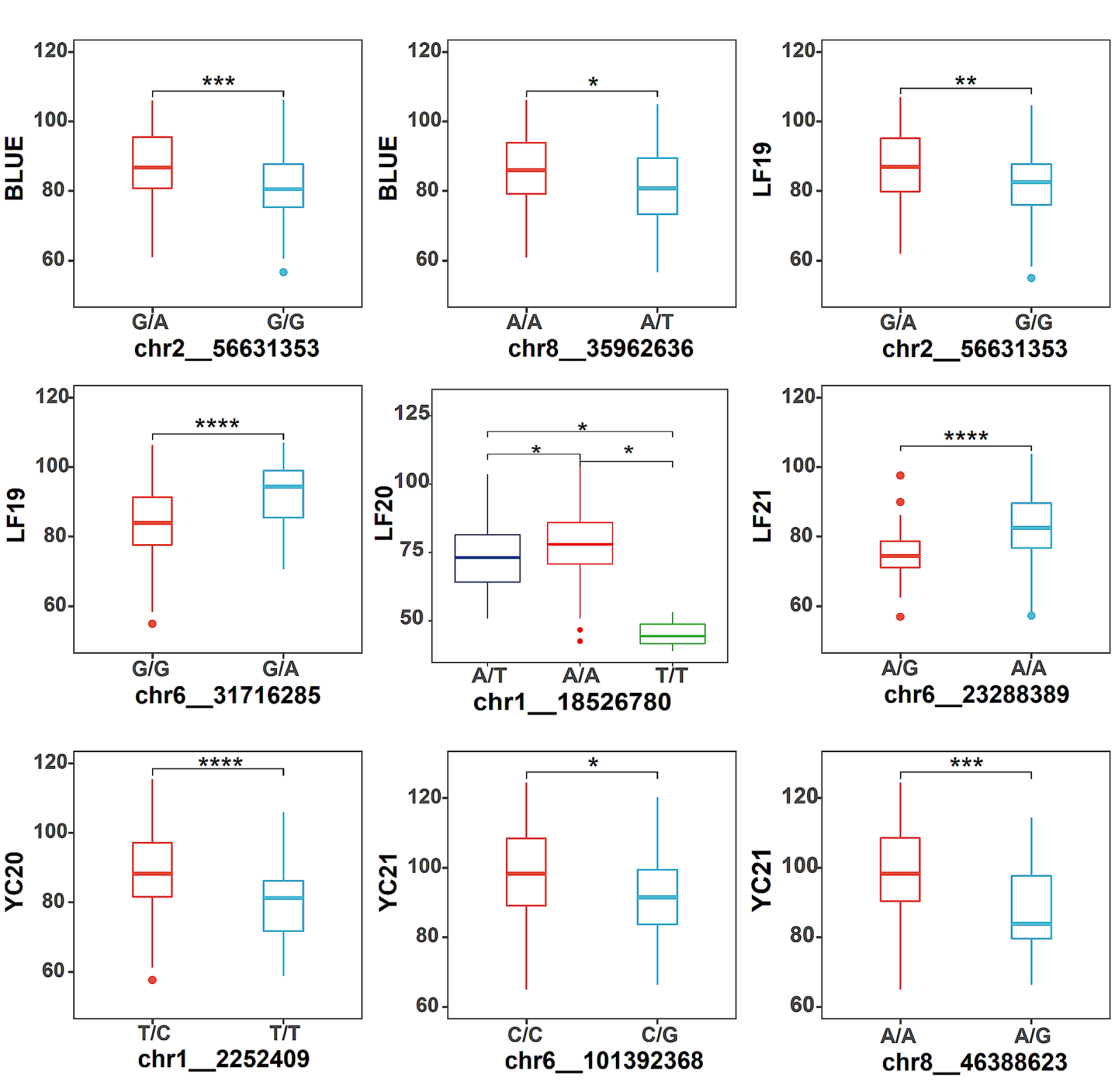
Haplotype analysis of significant SNPs. The lead SNPs were selected as the most significant SNPs in each QTL interval (within 1 CM). Haplotype differences were tested by t-test. (*P < 0.05, *P < 0.01, ***P < 0.001, ****P < 0.0001)

To further validate the accuracy of our GWAS results and ascertain favorable haplotypes, haplotype analysis was performed for significant SNPs detected using the GLM model. The lead SNPs showed the most significant *P* value within one Mb interval, indicating the most robust association with alfalfa PH. Only the lead SNPs were used for haplotype analysis. All these lead SNPs (*chr1__18526780*, *chr1__2252409*, *chr2__56631353*, *chr6__101392368*, *chr6__23288389*, *chr6__31716285*, *chr8__35962636*, *chr8__46388623*) showed significant differences between the different haplotypes (Fig. 3). For example, *chr2__56631353*, which was identified using BLUE values, had two haplotypes (G/G and G/A). The PH of plants containing the G/A haplotype was significantly greater than that of plants with the G/G haplotype (*P* < 0.001). Another SNP identified by BLUE values, *chr8__35962636*, had two haplotypes (A/A and A/T). The PH of plants containing the A/A haplotype was greater than that of plants with A/T haplotype. For each significant SNP, the haplotype with greater PH was deemed favorable haplotype in this study.

**Fig. 4:**
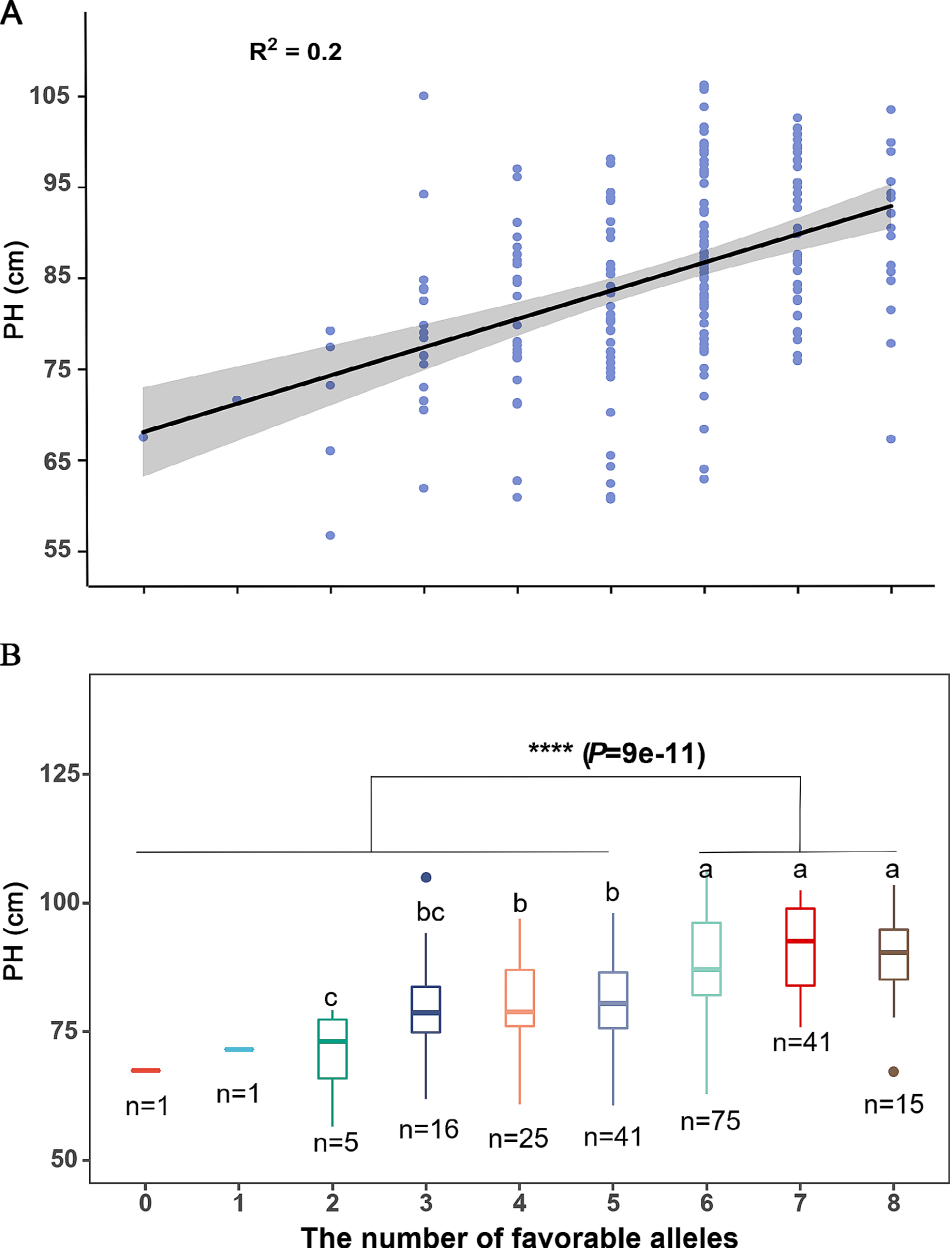
The relationship between the number of favorable genotypes and plant height (BLUE). **A** displays a scatter plot of the number of favorable genotypes and plant height, with the regression line and the correlation coefficient. **B** represents a box plot of plant height for different groups of accessions based on the number of favorable genotypes. Different letters above the box plots in **B** indicate significant differences among groups at the *P* < 0.05 level using Fisher’s least significant difference test. Accessions with six or more favorable genotypes had significantly greater plant height than other accessions (t-test, *P* = 9e^− 11^)

Next, the correlation between PH and the number of favorable genotypes carried by each accession in the association panel was calculated to determine the potential application of these favorable haplotypes in alfalfa PH breeding. The results showed a significant positive correlation between PH and the number of favorable genotypes, with a correlation coefficient of 0.2 (Fig. 4A). For other words, as the number of favorable genotypes increased, the height of the alfalfa plants tended to increase. The average heights of plants with two to eight favorable genotypes were 70.5, 79.78, 80.56, 80.82, 87.91, 90.95, and 89.44 cm, respectively. In our association panel, plants with six or more favorable genotypes were significantly taller than those with fewer than six favorable genotypes (t-test, *P* = 9e^− 11^, Fig. 4B). Overall, these findings demonstrate that our GWAS results are reliable. And alfalfa plants with more favorable haplotypes showed greater PH, indicating that the SNPs identified in our study can be used for genetic improvement of the alfalfa PH breeding program.

Additionally, the distribution of favorable haplotypes for each SNP in different subgroups of plants was calculated (Table S3). The results showed that the proportion of favorable genotypes in plants increased during the breeding process from wild to cultivars for three SNPs (*chr2__56631353*, *chr8__46388623*, and *chr8__35962636*), whereas it decreased for three other SNPs (*chr1__18526780*, *chr6__23288389*, and *chr6__31716285*) (Table S3). According to geographical origin, the proportion of favorable genotypes for the four SNPs (excluding *chr1__18526780*, *chr6__23288389*, *chr6__31716285*, and *chr8__46388623*) did not differ significantly among different subgroups.

**Fig. 5:**
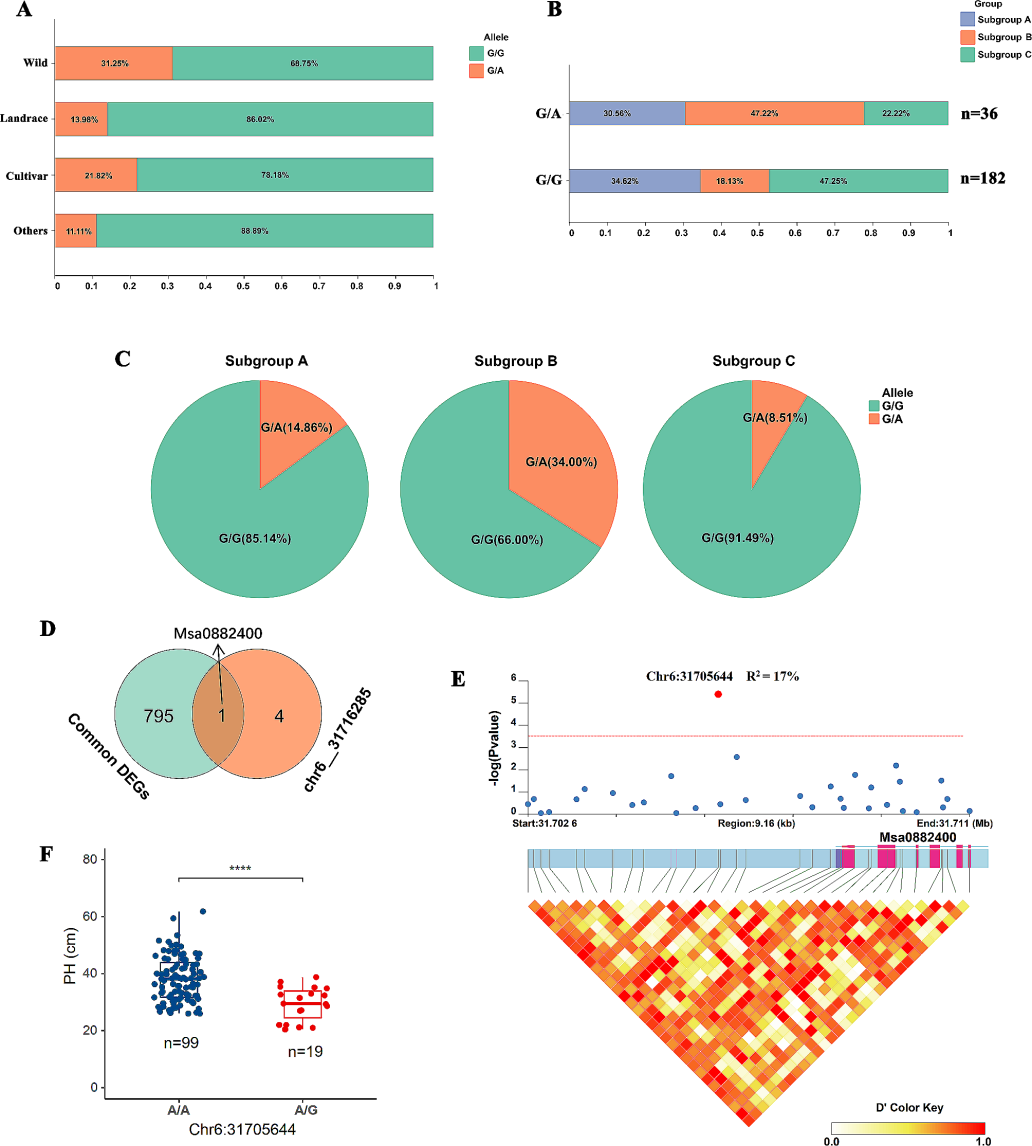
Characterization of the SNP *chr6__31716285* associated with plant height. **A** shows the genotype frequency of the SNP *chr6__31716285* (G/G and G/A) in four subgroups based on the improvement status (wild, landrace, cultivar, and other). **B** displays the geographical distribution of the accessions carrying the two genotypes of the SNP *chr6__31716285*. **C** presents the genotype frequency of the SNP *chr6__31716285* in three subgroups based on geographical origin (subgroup A, subgroup B, and subgroup C). Two accessions with missing genotypes at this locus were excluded from the analysis, resulting in a total of 218 accessions. **D** shows a Venn diagram illustrating the overlap between differentially expressed genes (DEGs) and genes within 20 kb upstream and downstream of the SNP *chr6__31716285*. **E** displays the gene structure of *Msa0882400* and association analysis of the SNPs within the gene region with plant height. The red dot indicates the significant SNP associated with plant height. **F** demonstrates the effect of the SNP haplotype of *Chr6:31705644* on plant height. The asterisks indicate significant differences (t-test, ****P < 0.0001)

In our association population, the frequency of favorable genotypes for 87.5% (7/8) of SNPs ranged from 65 to 87.3% overall, but only 16.4% (36/220) for *chr6__31716285* (Table S3). According to the breeding status, the frequency of the favorable G/A genotype in wild, landrace, and cultivar plants was 31.25%, 13.98%, and 21.82%, respectively (Fig. 5A). Among the 36 plants containing the G/A genotype, subgroup B represented the largest proportion (47.22%) (Fig. 5B). Further analysis revealed that the proportions of G/A genotypes in subgroups A, B, and C were 14.86%, 34.00%, and 8.51%, respectively (Fig. 5C). Differences in G/A genotype frequencies among plants from different geographical regions suggest that the G/A genotype has undergone different selection pressures during the breeding process in China and European-American countries. Our results indicate that the SNP *chr6__31716285* has great potential for use in alfalfa breeding programs.

### Association of *Msa0882400* with plant height in alfalfa

Through RNA sequencing analysis, a large number of DEGs (ES Vs PES) in both *M. sativa* ssp. *sativa* and *M. sativa* ssp. *falcata* were identified (Fig. S1A). A total of 796 common DEGs were identified in both subspecies, including 350 up-regulated (higher expression in ES) and 446 down-regulated genes (higher expression in PES) (Fig. S1B). These DEGs were categorized into biological processes (BP), cellular components (CC), and molecular functions (MF) using GO enrichment analysis (Table S5). In the CC category, the DEGs were mainly enriched (*P* < 0.05) in terms such as “cell wall,” “external encapsulating structure,” “plant-type cell wall,” “microtubule associated complex,” “vacuole,” and “microtubule cytoskeleton” (Table S5, Fig. S1C). In the BP category, the DEGs were mainly enriched (*P* < 0.05) in terms such as “secondary metabolite biosynthetic process,” “cell wall organization or biogenesis,” “cutin biosynthetic process,” and “plant-type cell wall organization or biogenesis” (Table S5, Fig. S1D). Furthermore, these DEGs were involved in processes such as “lignin biosynthetic process,” “lignin metabolic process,” “multidimensional cell growth,” “suberin biosynthetic process,” “plant-type primary cell wall biogenesis,” “cell wall modification,” “response to salicylic acid,” “cellulose biosynthetic process,” “cellulose metabolic process,” “response to ethylene,” “response to gibberellin,” “reproductive shoot system development,” and “gibberellin biosynthetic process” (Table S5). According to the results of the GO enrichment analysis, these differentially expressed genes can affect PH, as they are significantly associated with plant growth and development, particularly cell wall synthesis.

The frequency of the favorable genotype of chr6__31716285 was only 16.4%, so it had great potential for use in alfalfa PH improvement breeding. Five genes were located within 20 kb upstream and downstream of this marker. Among them, the expression of *Msa0882400* differed significantly between ES and PES in both M. sativa ssp. sativa and M. sativa ssp. falcata (Fig. 5D). *Msa0882400* encodes a mitochondrial phosphate carrier protein 3, which is highly similar to *Arabidopsis* phosphate transporter 3;1 (PHT3;1). In addition, according to our candidate gene association analysis, *Chr6:31705644*, located 2,440 bp upstream of *Msa0882400*, was significantly associated with PH (*P* = 4.03e^− 06^, R^2^ = 17%) (Fig. 5E). Alfalfa plants with the A/A genotype at this locus were significantly taller than those with the A/G genotype (*P* < 0.0001) (Fig. 5F). This suggests that *Msa0882400* may play a role in determining the PH of alfalfa.

### Prediction of candidate genes

To identify potential genes associated with PH, the genes located within 200 kb upstream and downstream of significant SNPs were functionally annotated using BLASTP in EnsemblPlants (https://plants.ensembl.org/index.html). In addition to *Msa0882400*, a total of 21 genes were identified that could be related to alfalfa PH (Table 2). These genes are involved in various biological processes and pathways associated with plant growth. For example, three genes (*Msa0000770*, *Msa0000870*, and *Msa0000900*) near the SNP *chr1__2252409* were annotated as NF-YA4, ORA47, and STM, respectively. Another gene (*Msa0012470*) near the SNP *chr1__18526780* was annotated as the two-component response regulator *ARR11*. Seven genes within the *chr2__56631353* region were annotated as members of the cytochrome 72 subfamily A of the P450 family. A gene, *Msa0879960*, near the SNP *chr6__23288389*, was annotated as GCP4, a gamma-tubulin complex protein that mediates microtubule nucleation and organization. Within 200 kb upstream and downstream of the SNP chr6__101392368, six vacuolar protein sorting-associated protein 54 were annotated. On chromosome 8, three candidate genes were annotated: two near the SNP chr8__35962636 and one near the SNP chr8__46388623. These genes were annotated as AP2-like ethylene-responsive transcription factor BBM, growth-regulating factor 5, and transcription factor MYB124, respectively.

**Table 2:** Candidate genes for plant height inferred from SNP associations

SNP	GeneID	Position				BLAST-*P*			
		Chr.	Start_Pos	End_Pos	Stand	Gene hit	Protein_coding description	E-value	%ID
*chr1__2252409*	*Msa0000770*	group1_1	2,060,836	2,064,177	+	*NF-YA4*	Nuclear transcription factor Y subunit A-4	2E-27	67.1
	*Msa0000870*	group1_1	2,252,206	2,252,895	-	*ORA47*	Ethylene-responsive transcription factor ERF018	1.7E-31	80.0
	*Msa0000900*	group1_1	2,295,957	2,296,952	-	*STM*	Homeobox protein SHOOT MERISTEMLESS	3.1E-09	81.5
*chr1__18526780*	*Msa0012470*	group1_1	18,688,566	18,695,517	+	*ARR11*	Two-component response regulator ARR11	8.6E-51	65.9
*chr2__56631353*	*Msa0213500*	group2_1	56,507,524	56,509,864	-	*CYP72A14*	cytochrome P450, family 72, subfamily A, polypeptide 14	6.1E-103	67.6
	*Msa0213510*	group2_1	56,530,991	56,534,485	+	*CYP72A14*	cytochrome P450, family 72, subfamily A, polypeptide 14	6.1E-102	68.6
	*Msa0213530*	group2_1	56,645,118	56,649,493	+	*CYP72A14*	cytochrome P450, family 72, subfamily A, polypeptide 14	1.6E-100	67.1
	*Msa0213540*	group2_1	56,654,377	56,658,890	+	*CYP72A14*	cytochrome P450, family 72, subfamily A, polypeptide 14	1.5E-101	68.1
	*Msa0213550*	group2_1	56,669,876	56,673,915	+	*CYP72A14*	cytochrome P450, family 72, subfamily A, polypeptide 14	6.1E-103	67.6
	*Msa0213560*	group2_1	56,682,029	56,687,014	+	*CYP72A14*	cytochrome P450, family 72, subfamily A, polypeptide 14	1E-103	67.1
	*Msa0213570*	group2_1	56,690,608	56,695,959	+	*CYP72A15*	cytochrome P450, family 72, subfamily A, polypeptide 15	1.4E-98	69.2
*chr6__23288389*	*Msa0879960*	group6_1	23,154,329	23,154,966	-	*GCP4*	GAMMA-TUBULIN COMPLEX PROTEIN 4	3.4E-09	92.0
*chr6__31716285*	*Msa0882400*	group6_1	31,708,084	31,711,243	+	*PHT3;1*	Mitochondrial phosphate carrier protein 3, mitochondrial	1.60E-168	76.6
*chr6__101392368*	*Msa0902630*	group6_1	1.01E+08	1.01E+08	+	*VPS54*	Vacuolar protein sorting-associated protein 54, chloroplastic	1.5E-46	65.0
	*Msa0902640*	group6_1	1.01E+08	1.01E+08	-	*VPS54*	Vacuolar protein sorting-associated protein 54, chloroplastic	2.6E-58	57.3
	*Msa0902650*	group6_1	1.01E+08	1.01E+08	-	*VPS54*	Vacuolar protein sorting-associated protein 54, chloroplastic	9.2E-33	80.0
	*Msa0902660*	group6_1	1.01E+08	1.01E+08	+	*VPS54*	Vacuolar protein sorting-associated protein 54, chloroplastic	4.5E-32	80.0
	*Msa0902670*	group6_1	1.01E+08	1.01E+08	+	*VPS54*	Vacuolar protein sorting-associated protein 54, chloroplastic	1.4E-46	65.0
	*Msa0902680*	group6_1	1.01E+08	1.01E+08	-	*VPS54*	Vacuolar protein sorting-associated protein 54, chloroplastic	8.4E-35	69.0
*chr8__35962636*	*Msa1191510*	group8_1	36,066,741	36,070,205	+	*BBM*	AP2-like ethylene-responsive transcription factor BBM	9.5E-38	85.1
	*Msa1191520*	group8_1	36,107,465	36,109,316	-	*GRF5*	Growth-regulating factor 5	6.8E-62	70.9
*chr8__46388623*	*Msa1196690*	group8_1	46,306,239	46,316,347	-	*MYB124*	Transcription factor MYB124	9.8E-32	78.1

## Discussion

PH is a quantitative trait that can be easily measured and correlates significantly with yield, making it an important phenotypic trait for selection in breeding programs [3, 6]. This is particularly true for forage crops such as alfalfa, harvested from their aboveground stems and leaves rather than from their reproductive parts. In alfalfa, the correlation between PH and yield can reach 0.79 [24]. As a result, considerable efforts have been devoted to uncovering the genetic basis of PH in this species. Previous studies have identified markers that are significantly associated with PH in specific genetic backgrounds using biparental [19, 24, 35] or natural association mapping populations [20, 22, 25]. However, few QTLs or SNPs have been validated for different genetic backgrounds. PH was measured in a diverse panel of 220 alfalfa varieties in multiple environments and identified several SNPs associated with this trait using GWAS. However, the SNPs identified in this study differed from those reported in previous studies [25]. One reason for this may be that our study only identified variants within the association population.

Pyramid breeding, which involves aggregating favorable genotypes for complex quantitative traits, has been used in crop breeding to develop superior varieties [36, 37]. For example, a high yielding and drought-tolerant rice line (MR219) was developed using marker-assisted breeding in pyramid QTLs related to drought tolerance, resulting in a yield advantage of ≥ 1500 kg/ha [38]. Similarly, three inbred rice lines with enhanced tolerance to dehydration, salinity, and submergence stress were developed by introgressing main-effect QTLs conferring tolerance to drought (*qDTY1.1*, *qDTY2.1*), salinity (*Saltol*), and submergence (*Sub1*) using a marker-assisted backcross breeding approach. These lines exhibited better performance than the recurrent parent [39]. Pyramid QTLs controlling agronomic traits have also been widely used in crops such as wheat [40, 41], and maize [42, 43]. Our study identified significant phenotypic differences between the favorable and alternative genotypes of the eight SNPs. These findings will facilitate the pyramiding of favorable genotypes in new alfalfa varieties through breeding programs. But it should be noted that increasing the pH can effectively increase biomass, but it will also increase the risk of lodging. Therefore, while increasing plant height, it is also necessary to increase stem diameter. At the same time, using the significant SNPs identified in this study, varieties with different plant heights can be selected according to different planting areas. For instance, it is prudent to choose cultivars with lower PH in windy planting regions (clustering less favorable genotypes) to minimize lodging.

Our study identified a phosphate transporter gene, *Msa0882400*, near the marker *chr6__31716285*. This gene is highly homologous to *Arabidopsis* phosphate transporter 3;1. Through a gene-based association analysis, it has been confirmed that *Msa0882400* is associated with PH in alfalfa. Phosphorus is a macroelement required for plant growth, and its availability is one of the key factors limiting plant growth and development [44]. Phosphate transporters (PTs) play an essential role in the uptake, transport, and remobilization of phosphorus in plants [45, 46]. There is strong evidence that overexpression of phosphate transporter genes can enhance phosphorus uptake and improve plant growth and development in low phosphorus environments. For example, the overexpression of *OsPT6* in rice resulted in a significant increase in PH [47]. Similarly, *OsPT6* overexpression in transgenic vegetable soybean lines improved phosphorus accumulation, growth efficiency, and PH under low phosphorus stress [48]. In chrysanthemums, transgenic plants constitutively expressing *CmPT1* grew taller than non-transformed wild-type plants under both phosphorus-sufficient and phosphorus-deficient conditions [49]. Other studies have shown that phosphate transporters regulate plant responses to drought and salt stress. Therefore, it is likely that *Msa0882400* plays a crucial role in the growth, development, and stress response of alfalfa.

In addition to *Msa0882400*, 21 additional genes were identified as potentially linked with plant growth and development between 200 kb upstream and downstream of significant SNPs identified in more than one GWAS model. Notably, seven tandem duplications of cytochrome P450 genes (CYP72A) and six duplications of vacuolar protein sorting-associated protein 54 (VPS54) genes were found near the markers *chr2__56631353* and *chr6__101392368*, respectively (Table 2). The cytochrome P450 superfamily of genes is involved in various aspects of plant growth, development, and stress resistance [50]. For example, in *Arabidopsis*, the CYP72A9 protein converts the highly biologically active molecule 13-H GA into the less biologically active molecule 13-OH GA. This conversion reduces the levels of bioactive gibberellins in plants overexpressing *CYP72A9*, resulting in a semi-dwarf phenotype [51]. VPS54 produced subunits of the vacuolar protein sorting-associated protein 54, a key component of the golgi-associated retrograde protein (GARP) complex. Defects in any of the GARP components have been shown to affect development in mice [52], *Caenorhabditis elegans* [53], and *Arabidopsis* [54], suggesting that the GARP complex was conserved across species. For example, the *Arabidopsis UNHINGED* gene encoded a homolog of VPS51 and plays a role in leaf development [54]. Several transcription factors related to plant growth and development were also identified, including *NF-YA4* (*Msa0000770*), *ORA47* (*Msa0000870*), *ARR11* (*Msa0012470*), and *MYB124* (*Msa1196690*). These candidate genes may be used for further functional validation to confirm their roles in regulating PH in alfalfa.

The ability to associate markers with phenotypes depends on the variation attributed to quantitative trait nucleotides and the number of individuals in the population [55]. In our study, an association analysis of PH was conducted in alfalfa and identified eight significant SNPs and 22 potential candidate genes associated with this trait. Therefore, future studies should consider increasing the population size and the amount of variation related to PH within the mapping population to identify more major SNPs or genes associated with this trait. Both the number and the length of the internodes have been reported to affect the height of alfalfa. Furthermore, branches with more internodes typically have more leaves and a higher leaf-to-stem ratio [56], and may be of better quality. Therefore, future studies on PH can also focus on the number and length of the internodes in alfalfa.

## Conclusion

In our study, GWAS was performed of PH variation in *M. sativa*, an important forage crop, using a diverse panel of 220 accessions of different origins and improvement statuses. Eight SNPs significantly associated with PH were identified, explaining 8.59–12.27% of the phenotypic variance. 22 candidate genes within the associated regions were annotated, including Msa0882400, which encoded a phosphate transporter and was validated through a transcriptome and candidate gene association analysis. The markers identified in our study can be utilized for MAS to expedite the development of new alfalfa cultivars with adjusted plant heights. Additionally, with further investigation, our results can offer new insights into the genetic and molecular mechanisms responsible for PH variation in alfalfa.

### Electronic supplementary material

Below is the link to the electronic supplementary material.

Supplementary Material 1

Supplementary Material 2

Supplementary Material 3

Supplementary Material 4

Supplementary Material 5

Supplementary Material 6

Supplementary Material 7

## Data Availability

All reasonable requests for data and research materials should be addressed to the corresponding author (kangjunmei@caas.cn). The raw RNA sequencing data used in this study were downloaded from the NCBI SRA database with the Bioproject accession numbers PRJNA276155. The raw sequence data of 220 alfalfa materials were upload to the National Genomics Data Center (NGDC, https://bigd.big.ac.cn/) under BioProject PRJCA004024. All raw sequence data of 118 individuals were upload to the National Genomics Data Center (NGDC, https://bigd.big.ac.cn/) under BioProject PRJCA018485.

## Abbreviations

PH: plant height
SNPs: single nucleotide polymorphisms
GWAS: genome-wide association studies
QTL: quantitative trait loci
LF: Langfang
YC: Yinchuan
BLUE: best linear unbiased estimator
H^2^: broad sense heritability
GLM: general linear model
MLM: mixed linear model
CMLM: compressed mixed linear model
PCA: principal component
K: kinship
ES: elongation internodes
PES: post-elongation internodes
DEG: differentially expressed genes
PVE: phenotypic variance explained
BP: biological processes
CC: cellular components
MF: molecular functions
PHT3;1: phosphate transporter 3;1
PTs: Phosphate transporters
