# Supplementary figures and images for: A genome-wide association study reveals novel loci and candidate genes associated with plant height variation in Medicago sativa
Source: BMC Plant Biol. 2024 Jun 13;24:544. doi: 10.1186/s12870-024-05151-z (PMC11177520; doi:10.1186/s12870-024-05151-z)

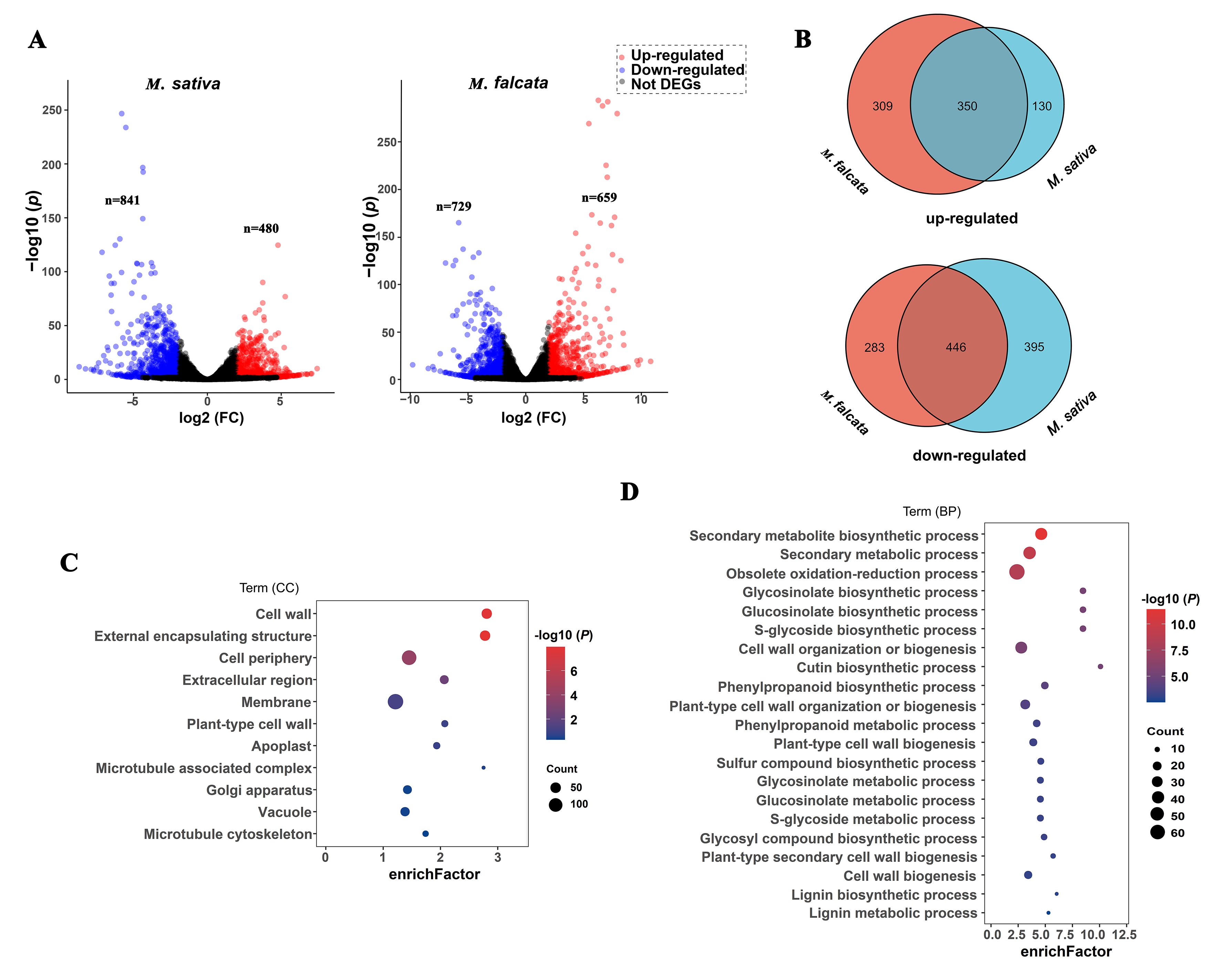

Supplement: Supplementary file 1 — Supplementary Material 1 [file 12870_2024_5151_MOESM1_ESM.tif]
