# Supplementary material for: A genome-wide association study reveals novel loci and candidate genes associated with plant height variation in Medicago sativa
Source: BMC Plant Biol. 2024 Jun 13;24:544. doi: 10.1186/s12870-024-05151-z (PMC11177520; doi:10.1186/s12870-024-05151-z)
Supplement: Supplementary file 7 — Supplementary Material 7 [file 12870_2024_5151_MOESM7_ESM.docx]

**A genome-wide association study reveals novel loci and candidate genes associated with plant height variation in *Medicago sativa***

Xueqian Jiang^1†^, Tianhui Yang^4,3†^, Fei He^1^, Fan Zhang^1^, Xu Jiang^1^, Chuan Wang^3^, Ting Gao^3^, Ruicai Long, Mingna Li, Qingchuan Yang^1^, Yue Wang^5^, Tiejun Zhang^2*^, Junmei Kang^1*^

**Supplementary Information**

**Fig. S1.** Comparison of the transcriptome of *M. sativa* ssp. *sativa* and *M. sativa* ssp. *falcata*. A presents a volcano plot of the differentially expressed genes (DEGs) between the two subspecies. B displays a Venn diagram illustrating the overlap of DEGs among the two subspecies. C and D show GO enrichment analysis for the DEGs.

**Table S1** Summary information for the 220 alfalfa accessions.

**Table S2** Phenotypic variation of plant height in the association panel.

**Table S3** Genotype frequency of favorable genotypes for plant height in different subgroups.

**Table S4** Information about the 33 SNPs.

**Table S5** GO enrichment for 796 differentially expressed genes.
